# Supplementary material for: Towards development of a high-strength stainless Mg alloy with Al-assisted growth of passive film
Source: Nat Commun. 2022 Oct 3;13:5838. doi: 10.1038/s41467-022-33480-w (PMC9529920; doi:10.1038/s41467-022-33480-w)
Supplement: Supplementary file 1 — Supplementary information [file 41467_2022_33480_MOESM1_ESM.pdf]

Supplementary Information

Towards development of a high-strength stainless Mg alloy with Al-assisted growth of passive film

Qingchun Zhu[1], Yangxin Li[1,*], Fuyong Cao[2], Dong Qiu[3], Yao Yang[1], Jingya Wang[1], Huan Zhang[1], Tao Ying[1], Wenjiang Ding[1], Xiaoqin Zeng[1,*]

[1]National Engineering Research Center of Light Alloy Net Forming and State Key Laboratory of Metal Matrix Composite, Shanghai Jiao Tong University, Shanghai, PR China

[2]Center for Marine Materials Corrosion and Protection College of Materials, Xiamen University, Xiamen, PR China

[3]Centre for Additive Manufacturing, School of Engineering, RMIT University, Melbourne, Victoria, Australia

*Corresponding authors: Yangxin Li (astatium@sjtu.edu.cn), Xiaoqin Zeng (xqzeng@sjtu.edu.cn)

The PDF file includes:

Supplementary Notes

Supplementary Fig. 1 Microstructure regulation of the WA111 alloy.

Supplementary Fig. 2 Corrosion rate against volume fraction of LPSO phase in the WA111 alloy.

Supplementary Fig. 3 Potentials of the Al_2Y and LPSO phases in the WA111 alloy.

Supplementary Fig. 4 Mechanical properties and microstructures of the WA111 samples.

Supplementary Fig. 5 Schmid factor distribution histograms of WA111 T4-EX-1 sample.

Supplementary Fig. 6 TEM observation of the WA111 T4-EX-1sample before tensile test.

Supplementary Fig. 7 TEM observation of dislocations in the WA111 samples after tensile tests.

Supplementary Fig. 8 Salt spray tests of the WA111 and AZ91D alloys.

Supplementary Fig. 9 Microscopic surface morphologies of the WA111 alloy with different states after soaking in the 3.5 wt. % NaCl solution for 14 days without the corrosion products.

Supplementary Fig. 10 Quasi in-situ observation of the second phase shedding in the as-cast WA111 alloy before and after brine immersion.

Supplementary Fig. 11 EPMA results of corrosion product films in the as-cast W11 and WA111 alloys.

Supplementary Fig. 12 Morphology and element characterization of corrosion product film of the as-cast WA111 alloy after soaking in 3.5 wt. % NaCl solution for 1 hour.

Supplementary Fig. 13 XRD patterns of corrosion products of the as-cast WA111 alloy surface.

Supplementary Fig. 14 Equivalent circuit diagrams of the W11 and WA111 alloys.

Supplementary Fig. 15 Hydrogen volume evolution and thickness evolution of corrosion product film against immersion time in the WA111 alloy.

Supplementary Fig. 16 Surface morphology of W11 and WA111 alloys after soaking for a period of time.

Supplementary Fig. 17 The statistical diagram of mechanical and corrosion properties of magnesium alloys prepared by conventional methods with corresponding literature sources for Fig. 1f.

Supplementary References

Supplementary Notes

1. Microstructure and corrosion resistance of the WA111 alloy

Supplementary Fig. 1 shows representative microstructures and corresponding second phase identification in the WA111 alloy with different thermal mechanical treatments. Via comparing the morphologies and the EDS results of the second phases with those in literature[1, 2, 3], two main second phases, including the polygonal Al_2Y and lamellar LPSO, were identified in this system. Unlike the amount and distribution of Al_2Y particles that is hard to be adjusted after casting, the amount and distribution of LPSO phase is tunable under different heat treatment and thermal deformation conditions in the WA111 alloy. The volume fractions of the LPSO phase in the 520-states (including T4-1 and T4-EX-1) and the 550 states (including T4-2 and T4-EX-2) were 6.5 % and 17.0 %, respectively.

Although the amount and distribution of LPSO phase varies in these five different states, the corrosion rate remains at a fairly constant level, i.e. 0.16-0.25 mm y^{-1}, as shown in Supplementary Fig. 2 and Fig 1a. Via analyzing the volta-potential differences between the second phases and the surrounding matrix in the WA111 alloy, it was found that the volta-potential of Al_2Y and LPSO phases were 300 mV and 400 mV higher than the surrounding matrix, as respectively shown in Supplementary Fig. 3. The results indicate that these two second phases are both cathode phases, which cause the strong galvanic-corrosion only in the initial period of the immersion test. As a result, the sample T4-2 (550°C-16 h) with a lower volume fraction of LPSO phase (6.5%) exhibited a slightly lower corrosion rate (0.19 mm y^{-1}) than that of sample T4-1 (520°C-8h, 0.21 mm y^{-1}) with a higher volume fraction of LPSO phase (17%), as shown in Supplementary Fig. 2. However, the galvanic corrosion between the LPSO phase and Mg matrix is soon inhibited significantly through the formation of protective corrosion product film. Therefore, the corrosion rate shows a low sensitivity to the volume fraction LPSO phase in WA111 alloy. After the surface protective film is formed in 1200 s, as shown in Supplementary Fig. 16, the galvanic corrosion between the second phases and the surrounding Mg matrix is strongly inhibited.

2. Mechanical properties of the WA111 alloy

Supplementary Fig. 4a presents typical stress-strain curves upon tension of the T4-1 and T4-EX-1 WA111 samples. The yield strength of WA111 alloy after solution treatment at 520 °C for 8 hours (T4-1) is 160 MPa. After extrusion at 350 °C, the yield strength was increased dramatically to 350 MPa (T4-EX-1). Generally speaking, the key factors contributing to the yield strength ($\sigma_{0.2}$) of a magnesium alloy mainly consist of the friction stress of pure Mg (σ_{Mg}), solid-solution strengthening (σ_{ss}), grain-refinement strengthening (σ_{gb}), precipitation strengthening (σ_{ppt}) as well as texture strengthening (σ_{tex}), which can be expressed via the following equation.

$$\sigma_{0.2} = \sigma_{Mg} + \sigma_{ss} + \sigma_{gb} + \sigma_{ppt} + \sigma_{tex}$$

The yield strength contribution of pure Mg in the Mg-Y based alloys is about 17 MPa [4]. The yield strength contribution of solid-solution is evaluated by $\sigma_{ss} = m(M_Y * c_Y^{\frac{1}{2}} + M_{Al} * c_{Al}^{\frac{1}{2}})$ [5], where m=4.5 is the Taylor factor, M_x is the potency factor for a specific solute x, and c_x is atomic percent of a specific solute x in the matrix. With respect to the WA111 alloy, $M_Y = 124.9$ MPa, $M_{Al} = 19.6$ MPa and the content of Y element in matrix of T4-1 state WA111 sample was measured as $c_Y = 1.7$ at.%, $c_{Al} = 0.3$ at.% by EDS. Then, the σ_{ss} is about 80 MPa. Since the major strengthening precipitate in the WA111 alloy is LPSO phase, and its volume fraction and distribution are characterized in Supplementary Fig. 1, the yield strength contribution of LPSO phase in undeformed WA111 alloy can be evaluated by $\sigma_{ppt} = \Delta\tau_{basal} = \frac{Gb}{2\pi\sqrt{1-\nu}\left(\frac{0.953}{\sqrt{f_p}}-1\right)d_t} ln\frac{d_t}{b}$ [6], which is about 20 MPa. As seen in Supplementary Fig. 4 a-b, the T4-1 state WA111 sample, of which the yield strength is about 160 MPa, has an average grain size of ~40 μm with no obvious texture, the contribution of grain-refinement strengthening ($\sigma_{gb} = kd^{-\frac{1}{2}}$) is calculated to be about 43 (160-17-80-20) MPa. Then the Hall-Petch value k of WA111 alloy is estimated to be close to 252 MPa·μm$^{-1/2}$, which is one of the commonly used k value of Mg-Y based alloys in literature [7, 8].

After extrusion, the yield strength increment of the T4-EX-1 WA111 sample is

about 190 MPa. Since there is neither noticeable difference of solute concentration nor the volume fraction of secondary phases between T4 and T4-EX samples, the yield strength increment ($\Delta\sigma_{0.2}$) after extrusion was estimated to mainly come from the grain refinement ($\Delta\sigma_{gb}$) as well as texture strengthening ($\Delta\sigma_{tex}$). In this work, a modified Hall-Petch relationship below is usually used to describe the compounded effect of grain size and texture on the yield strength: $\sigma_{y(gb+tex)} = \frac{0.3}{m_t}\left(\sigma_0 + kd^{-\frac{1}{2}}\right)$ [9, 10], where m_t and k are the average Schmid factor and the Hall-Petch slope, respectively, and $\sigma_0 = \sigma_{Mg} + \sigma_{ss} + \sigma_{ppt}$. The average grain sizes in the T4-1 and T4-EX-1 samples are 40 μm and 2 μm (Supplementary Fig. 4b and 4c), respectively. The average Schmid factor m_t of T4-EX-1 sample is about 0.27 (Supplementary Fig. 5), and the Hall-Petch factor k is set as 252 MPa $\mu m^{-1/2}$. Therefore, the estimated yield strength increment $\Delta\sigma_{0.2}$ contributing from grain refinement and texture is about 170 MPa, with $\Delta\sigma_{gb} =$ 135 MPa and $\Delta\sigma_{tex} = 35$ MPa, respectively. The rest of yield strength increment (20 MPa out of 190 MPa) might be attributed to the residual forest dislocations because immediate water-quenching just after extrusion could prohibit some dislocations from recovery (as shown in Supplementary Fig. 6). On the other hand, some LPSO lamellae were bent away from basal plane and hence increased the hindrance of basal dislocations (also shown in Supplementary Fig. 6). These two factors could further enhance the yield strength by 20 MPa to 350 MPa.

The reasons for achieving a moderate elongation after extrusion (e.g. from 4 % of the T4-1 to 8% of the T4-EX-1) are threefold. Firstly, the reduction of grain size is beneficial on the ductility of magnesium[11]. Secondly, the severe stress concentration around Al_2Y particles in WA111 alloy is significantly alleviated by the grain refinement caused by the extrusion process, thus delaying the fracture of the alloy[12]. Last but not least, sufficient non-basal slip systems are activated in the extruded WA111 samples. The TEM observation shows the dislocation behavior in the T4-1 and T4-EX-1 WA111 samples, respectively (See Supplementary Fig. 7). The two-beam bright field image shows the dislocation in solution treated WA111 sample is mainly <*a*> type while a number of <*c*+*a*> dislocations are activated in extruded WA111 sample. It is well

recognized that the poor plasticity of magnesium alloys is mainly caused by the limited slip systems that can be activated at room temperature. The observation of <*c*+*a*> dislocations in extruded WA111 sample indicates that non-basal slip systems are successfully activated, which facilitates subsequent plastic deformation and hence increases the ductility of WA111 alloy.

The extrusion induced ductility of WA111 alloy can also be witnessed on the fractography. It reveals that the tensile fractography of solution treated WA111 sample features a large number of cleavage planes and many Al_2Y particles are exposed to the fracture surface (as shown in Supplementary Fig. 4f). This result indicates that the fracture of the solution treated WA111 sample is cleavage dominated and the crack is probably initiated around the hard and brittle Al_2Y particles. After extrusion, the fracture mode turns from cleavage dominated to dimple dominated (as shown in Supplementary Fig. 4g) due to substantial grain refinement. In addition, the severe stress concentration around Al_2Y particles in WA111 alloy is significantly alleviated by the grain refinement caused by the extrusion process, thus delaying the fracture of the alloy[12].

3. Equivalent circuit diagrams of the W11 and WA111 alloys

In order to explain the form of WA111 alloy corrosion, we simulated the electrochemical AC impedance behavior of WA111 alloy. The equivalent circuit diagram has been listed above. Here are the parameters used in the fitting. R_s is solution resistance, R_a is charge transfer resistance, R_L is inductor resistance and R_f is film resistance. We can notice that the R_f of as-cast WA111 alloy is 2968 $\Omega \bullet cm^2$, which is two orders of magnitude higher than that of as-cast W11 alloy. This indicates a remarkable protection from corrosion by film in the as-cast WA111 alloy. The LPSO amount of T4-1 sample is higher than that of as-cast sample, leading to a lower R_f of T4-1 sample than that of as-cast sample. With the extension of soaking time, the R_f of as-cast sample increases gradually. These phenomena are consistent with the corrosion rate performance (hydrogen volume evolution and weight loss) in the WA111 alloy.

4. The hydrogen volume evolution and the thickness of protective film.

Supplementary Fig. 15a shows hydrogen evolution curves against immersion time of three representative WA111 samples. It shows that the hydrogen evolution volume of the WA111 alloy in different states all increases slowly with immersion time. However, the difference of the hydrogen volume between three specimens is marginal, indicating that the corrosion resistance of WA111 alloy shows lower sensitivity to microstructure compared to other commonly used Mg-Al or Mg-RE alloys due to formation of the protective film layer on the alloy surface. Supplementary Fig. 15b shows the thickness of the protective film layer with the immersion time. The thickness evolution of the corrosion product film (Supplementary Fig. 15b) shows that the grow rate of the film slows down with immersion time, which is consistent with the hydrogen evolution curves (Supplementary Fig. 15a).

For kinetics, the protective film formed quickly in the WA111 alloy at the early stage as shown in Supplementary Fig. 16, which is consistent with the polarization curves in Fig. 4a. The thickness evolution of the corrosion product film (Supplementary Fig. 15b) shows that the grow rate of the film slows down with immersion time. More importantly, the EIS curves with different soaking time in Fig. 4c verify that the resistance of the WA111 increases significantly in the first 1-2 days and reaches a plateau till the end. In summary, the protective corrosion product film of WA111 forms instantly and soon covers the whole surface of the specimen in the immersion test. The corrosion resistance of film reaches a high level in 1-2 days and keeps stable till the end.

When the Al and Y solute atoms on the sample surface are in contact with the brine solution, they lost electrons and then transform into Al^{3+} and Y^{3+} ions, respectively, which dissolve into the solution simultaneously. In contrast, the Al and Y atoms in the second phases do not dissolve into the solution because the second phases are cathode phases, as shown in Supplementary Fig. 3. Due to the low solubility of Al^{3+} and Y^{3+}, their concentrations in the brine solution quickly reach saturation, leading to the precipitation of Al and Y hydroxides on the alloy surface and the subsequent formation of protective product film.

Supplementary Tables

Supplementary Table 1 The fitting results of the samples according to the equivalent circuit.

	R_s ($\Omega \cdot cm^2$)	Y_{0-f} ($\Omega \cdot s^n \cdot cm^{-2}$)	n_f	R_f ($\Omega \cdot cm^2$)	R_a ($\Omega \cdot cm^2$)	Y_{0-a} ($\Omega \cdot s^n \cdot cm^{-2}$)	n_a	R_L ($\Omega \cdot cm^2$)	L ($H \cdot cm^{-2}$)
W11-F	7	1.79×10^{-5}	0.97	14	106	1.09×10^{-5}	0.96	184	26
WA111-F-1.5h	7	1.33×10^{-5}	0.94	2968	2743	1.10×10^{-3}	0.69	—	—
WA111 -T4-1-1.5h	10	1.37×10^{-5}	0.93	2080	1447	1.20×10^{-3}	0.68	—	—
WA111- T4-EX-1-1.5h	15	1.71×10^{-5}	0.94	3161	2517	1.58×10^{-3}	0.63	—	—
WA111-F-1day	17	1.64×10^{-5}	0.94	4526	4810	0.85×10^{-3}	0.58	—	—
WA111-F-2day	8	1.80×10^{-5}	0.94	6311	5146	0.45×10^{-3}	0.66	—	—
WA111-F-3day	8	1.90×10^{-5}	0.94	7134	6418	0.41×10^{-3}	0.64	—	—
WA111-F-9day	8	2.85×10^{-5}	0.92	6755	6967	0.53×10^{-3}	0.62	—	—
WA111-F-14day	10	3.69×10^{-5}	0.91	7346	7051	0.68×10^{-3}	0.66	—	—

Supplementary Table 2 Chemical compositions of the alloys determined by ICP analysis.

Alloys (wt. %)	Y	Zn	Al	Fe	Ni	Cu	Si	Mg
WA111	11.00	/	0.91	0.018	<0.0005	<0.0005	0.29	Bal.
W11	11.41	/	0.036	0.020	<0.0005	<0.0005	0.32	Bal.
AZ91D	/	0.65	8.96	<0.001	<0.0005	<0.0005	<0.01	Bal.

Supplementary Figures

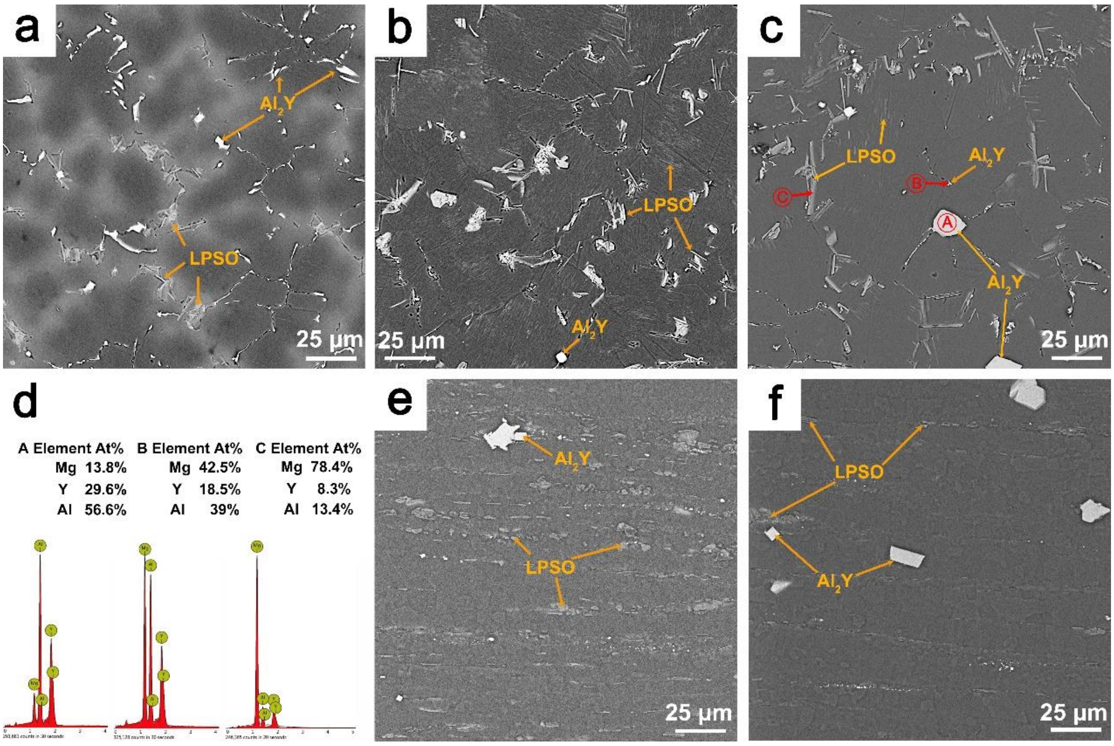

Supplementary Fig. 1 Microstructure of the WA111 alloy in different states. (a) as-cast state; (b) T4-1 state; (c) T4-2 state, with the EDS results of marked Al_2Y and LPSO particles shown in (d); (e) T4-EX-1 state; (f) T4-EX-2 state.

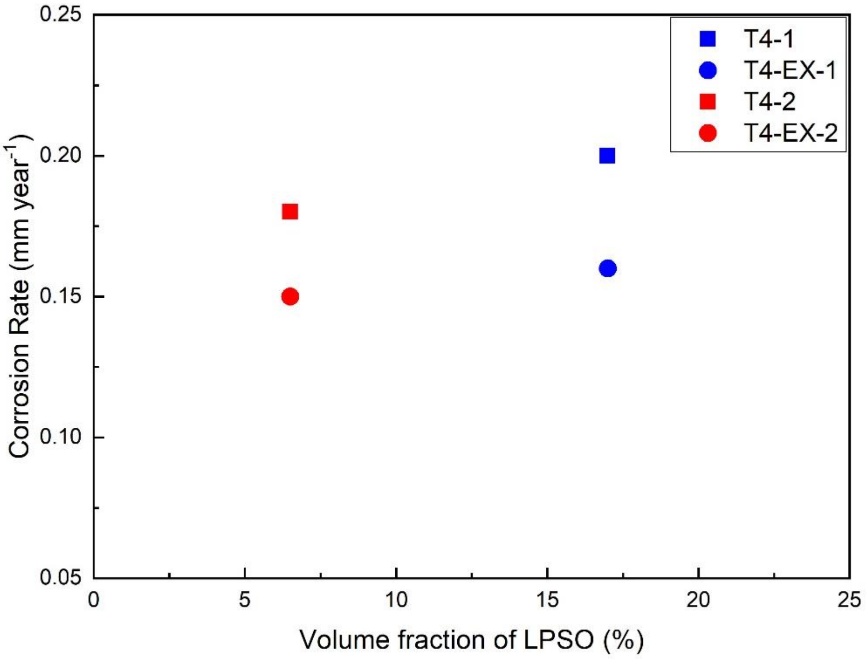

Supplementary Fig. 2 Corrosion rate against volume fraction of LPSO phase in WA111 alloy. The volume fractions of the LPSO phase in the 520-states (including T4-1 and T4-EX-1) and the 550 states (including T4-2 and T4-EX-2) were 6.5 % and 17.0 %, respectively. The volume fraction of LPSO phase has little effect on the corrosion rate of WA111 alloy.

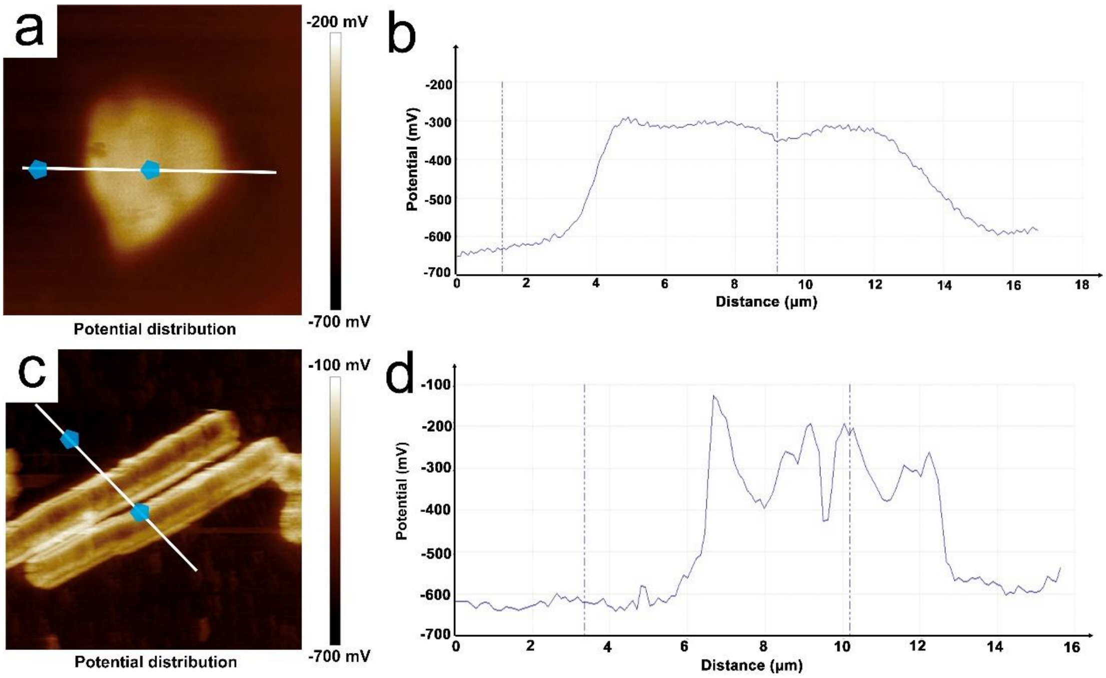

Supplementary Fig. 3 Potential analysis of second phases in the WA111 alloy. Potentials of the (a-b) Al_2Y and (c-d) LPSO phases in the WA111 alloy, respectively. The dotted lines in (b) and (d) represent the corresponding position of blue solid pentagon in (a) and (c), respectively.

.

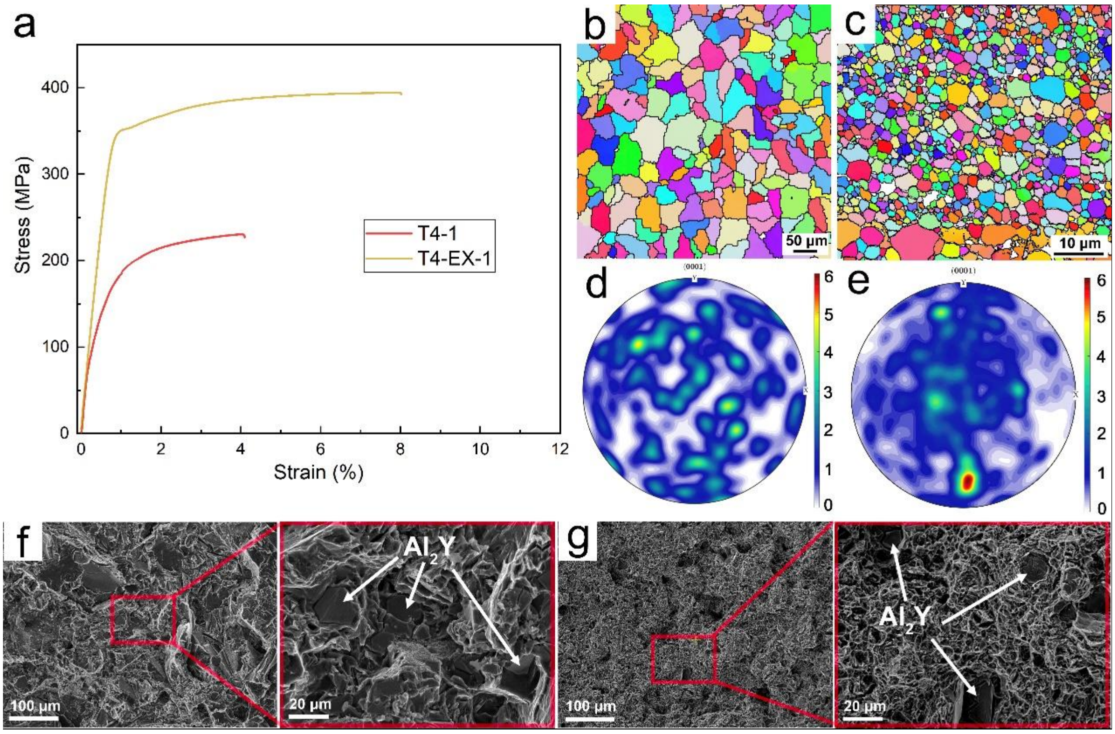

Supplementary Fig. 4 Mechanical properties and microstructures of the WA111 samples. (a) tensile stress-strain curves; EBSD maps and pole figures of T4-1 WA111 sample (b and d), T4-EX-1 WA111 sample (c and e), with the average grain sizes of 40 μm and 2 μm, respectively. (f) and (g) are the tensile fractographies of T4-1 and T4-EX-1 state WA111 samples, respectively.

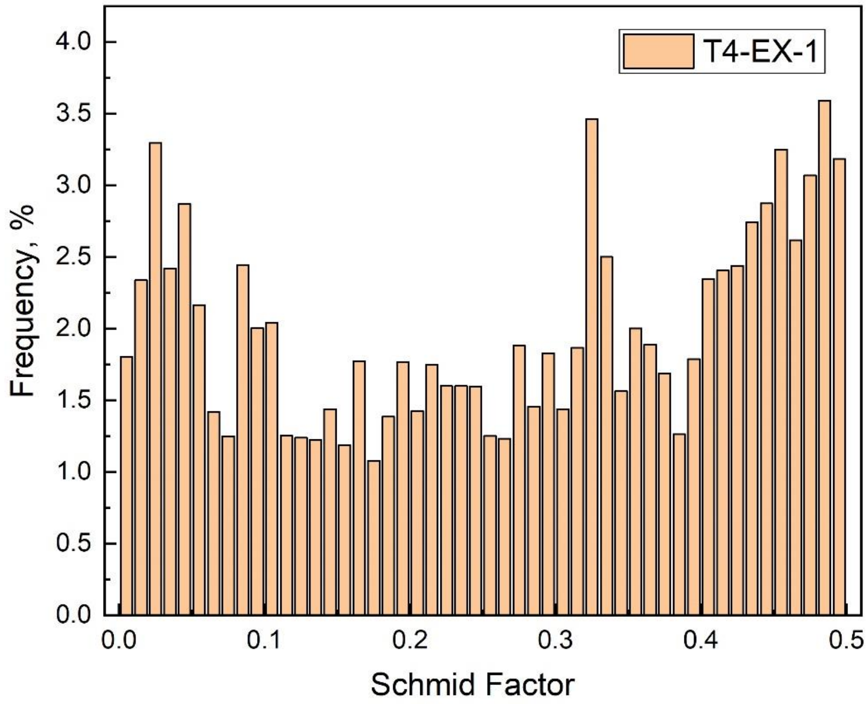

Supplementary Fig. 5 Schmid factor distribution histograms of T4-EX-1 WA111 sample. The average Schmid factor m_t of T4-EX-1 WA111 sample is about 0.27.

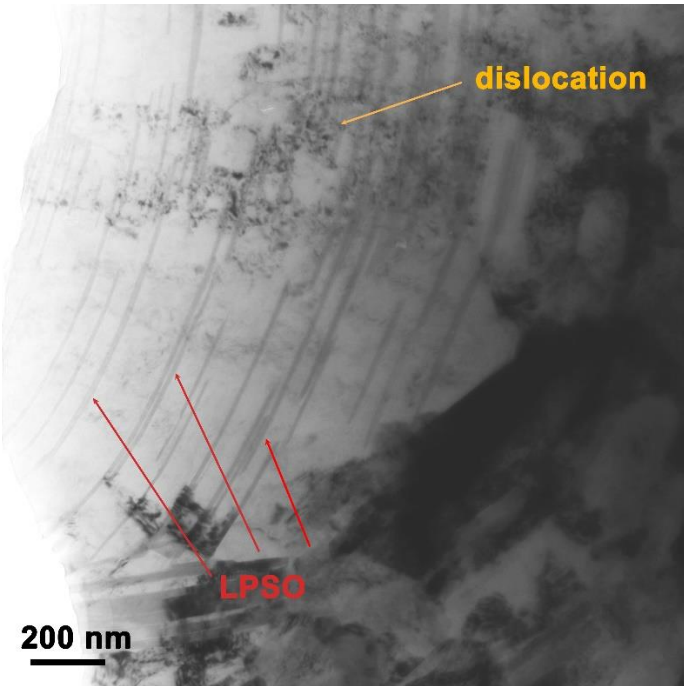

Supplementary Fig. 6 TEM observation of the T4-EX-1 WA111 sample before tensile test. Some LPSO lamellae were bent away from basal plane and hence increased the hindrance of basal dislocations. In addition, some residual dislocations caused by immediate water-quenching just after extrusion were observed.

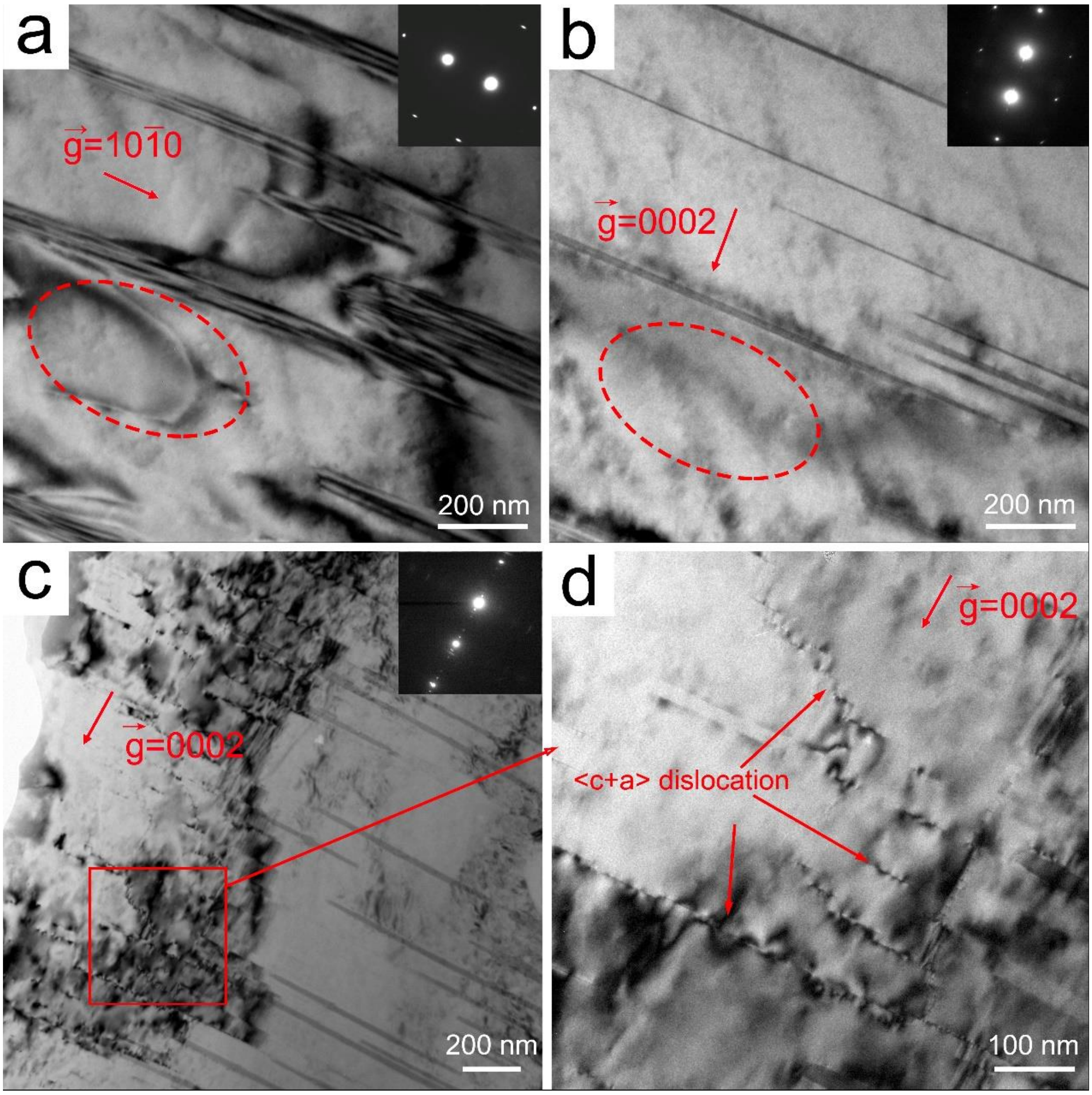

Supplementary Fig. 7 Bright field TEM images of statistically stored dislocations in the WA111 alloy after tensile test in two-beam conditions. (a) T4-1 sample with $\vec{g} = 10\bar{1}0$; (b) T4-1 sample with $\vec{g} = 0002$; (c) T4-EX-1 sample with $\vec{\boldsymbol{g}} = 0002$; (d) the enlarged portion of a local region in (c). The insets of the panels (a-c) are diffraction patterns. The ovals with red dashed lines in panels (a-b) represent the same position with different two-beam conditions, indicating that the dislocations are basal <a> dislocations.

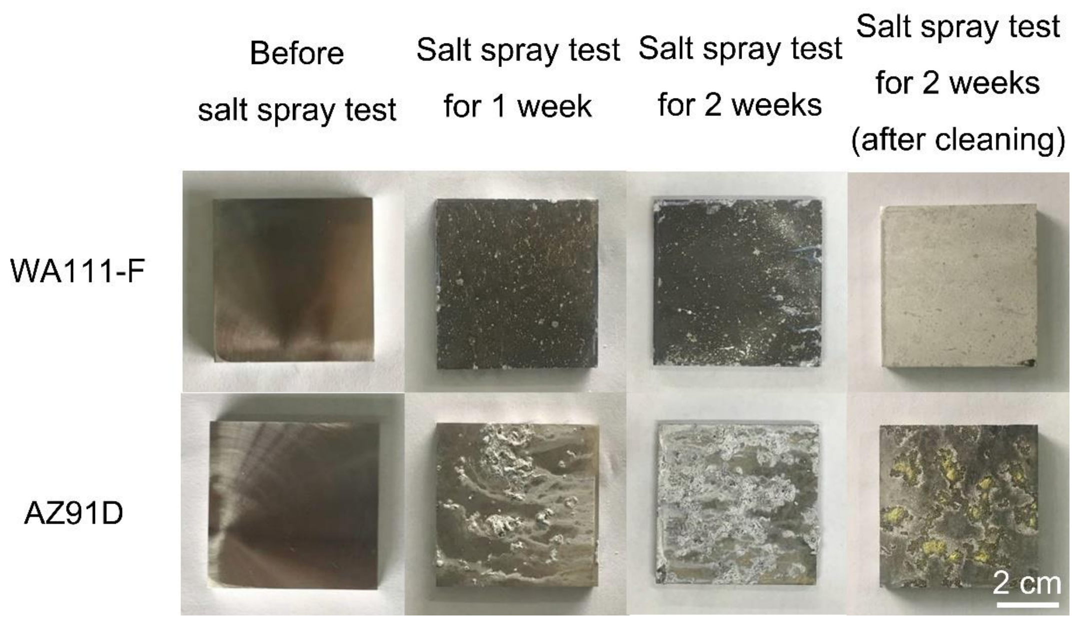

Supplementary Fig. 8 Salt spray tests of the WA111 and AZ91D alloys. The macroscopic corrosion of WA111-F alloy is very mild and uniform and no noticeable cavity was observed on the surface even after being salt sprayed for two weeks. However, the AZ91D alloy has been corroded much more substantially and there is serious pitting corrosion on the surface after salt spray test. Hence, the corrosion resistance of WA111 alloy is much better than AZ91D alloy.

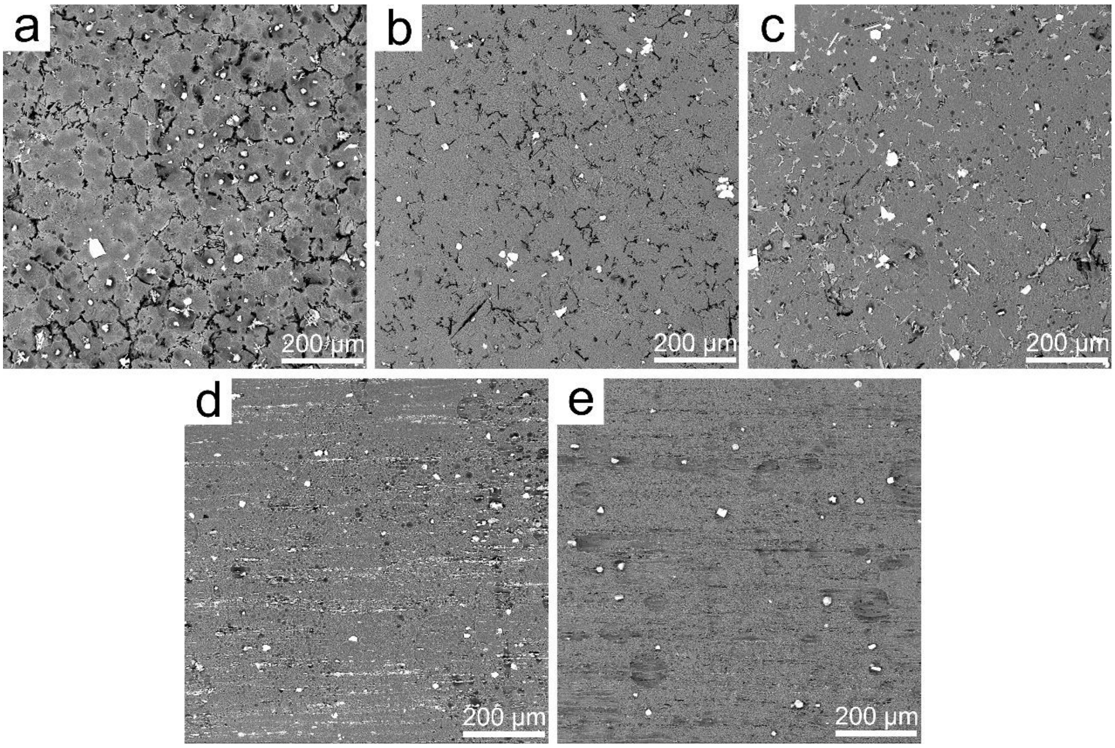

Supplementary Fig. 9 Microscopic surface morphologies of the WA111 alloy with different states after soaking in the 3.5 wt. % NaCl solution for 14 days without the corrosion products. (a) as-cast state; (b) T4-1; (c)T4-2; (d) T4-EX-1; (e) T4-EX-2.

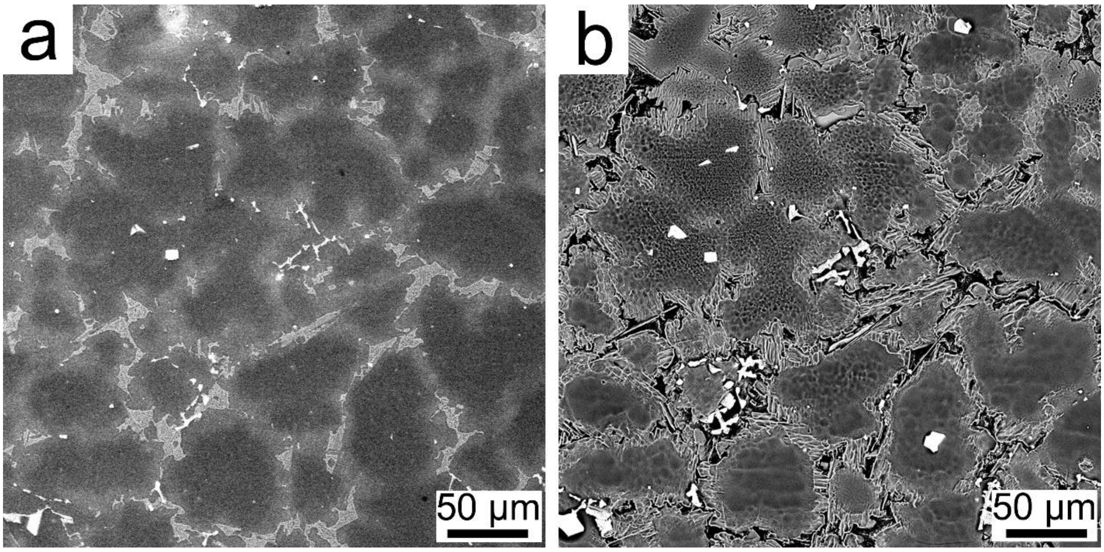

Supplementary Fig. 10 Quasi in-situ observation of the second phase shedding in the as-cast WA111 alloy before and after brine immersion. (a) microstructure before soaking in 3.5 wt. % NaCl solution; (b) microstructure of same position after soaking in 3.5 wt. % NaCl solution for 1 day.

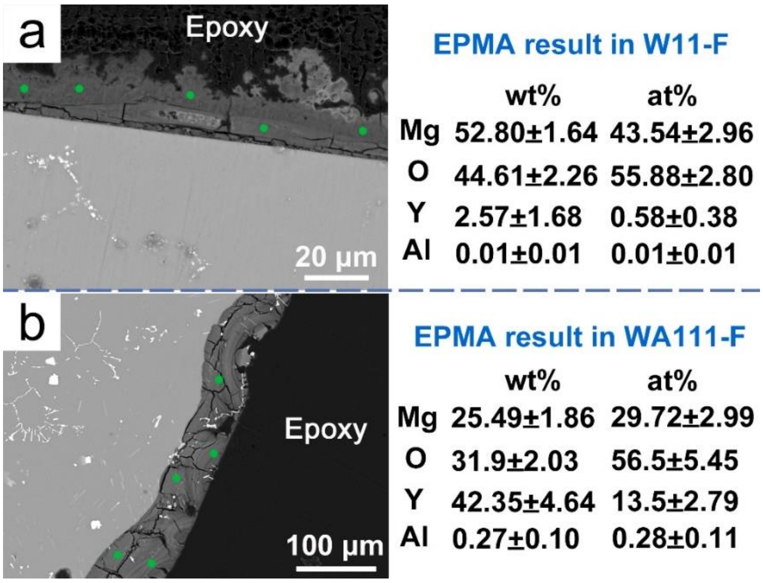

Supplementary Fig. 11 EPMA results of corrosion product films in the as-cast W11 and WA111 alloys after soaked in 3.5 wt. % NaCl solution. (a) as-cast W11 alloy soaked for 1 day; (b) as-cast WA111 alloy soaked for 14 days.

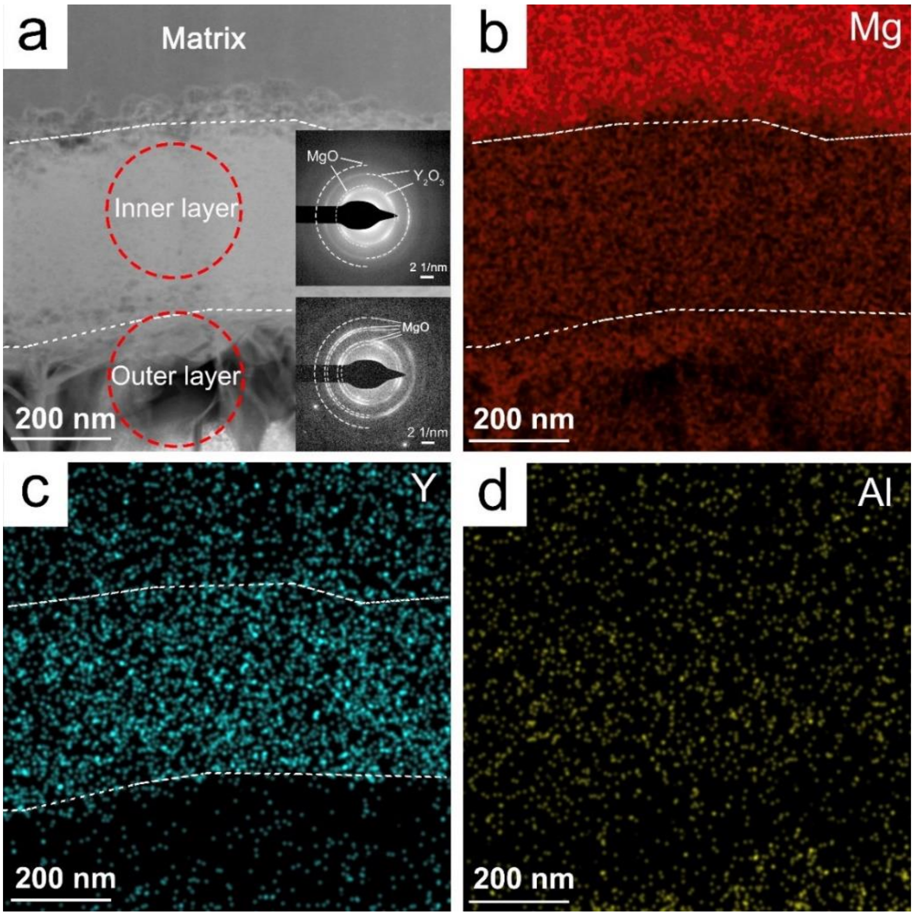

Supplementary Fig. 12 Morphology and element characterization of corrosion product film of the as-cast WA111 alloy after soaking in 3.5 wt. % NaCl solution for 1 hour. (a) HAADF-STEM image with SEAD patterns from inner layer (Y_2O_3) and outer layer (MgO); (b-d) corresponding EDS Mapping.

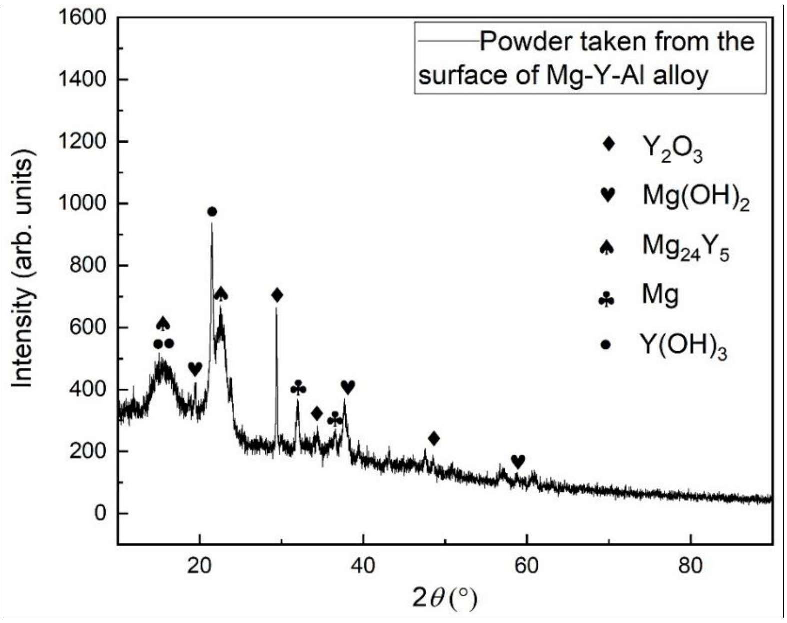

Supplementary Fig. 13 XRD patterns of corrosion products taken from the surface of as-cast WA111 alloy soaked for 14 days. A few strong diffraction peaks match well with Y_2O_3 and $Y(OH)_3$.

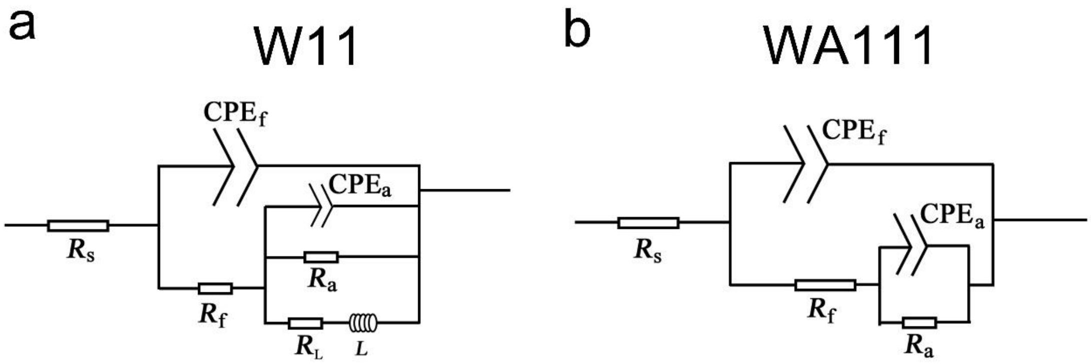

Supplementary Fig. 14 Equivalent circuit diagrams of the W11 and WA111 alloys. (a) as-cast W11 alloy; (b) WA111 alloy with different states.

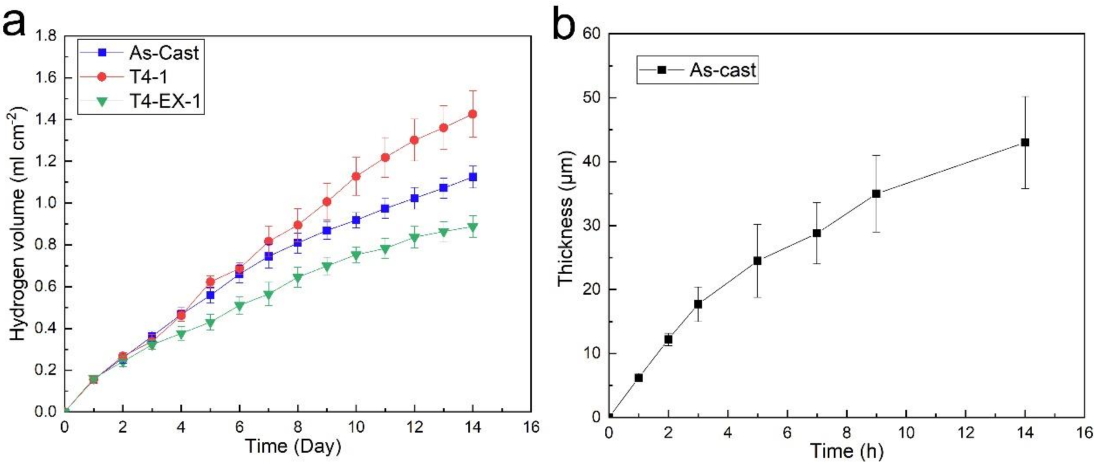

Supplementary Fig. 15 Kinetic description of corrosion process of WA111 alloy. (a) Hydrogen volume evolution and (b) thickness evolution of corrosion product film against immersion time in the WA111 alloy. The hydrogen evolution and corrosion film thickness of the alloy were calculated as the average of three samples, and the error bars were plotted according to their standard deviation.

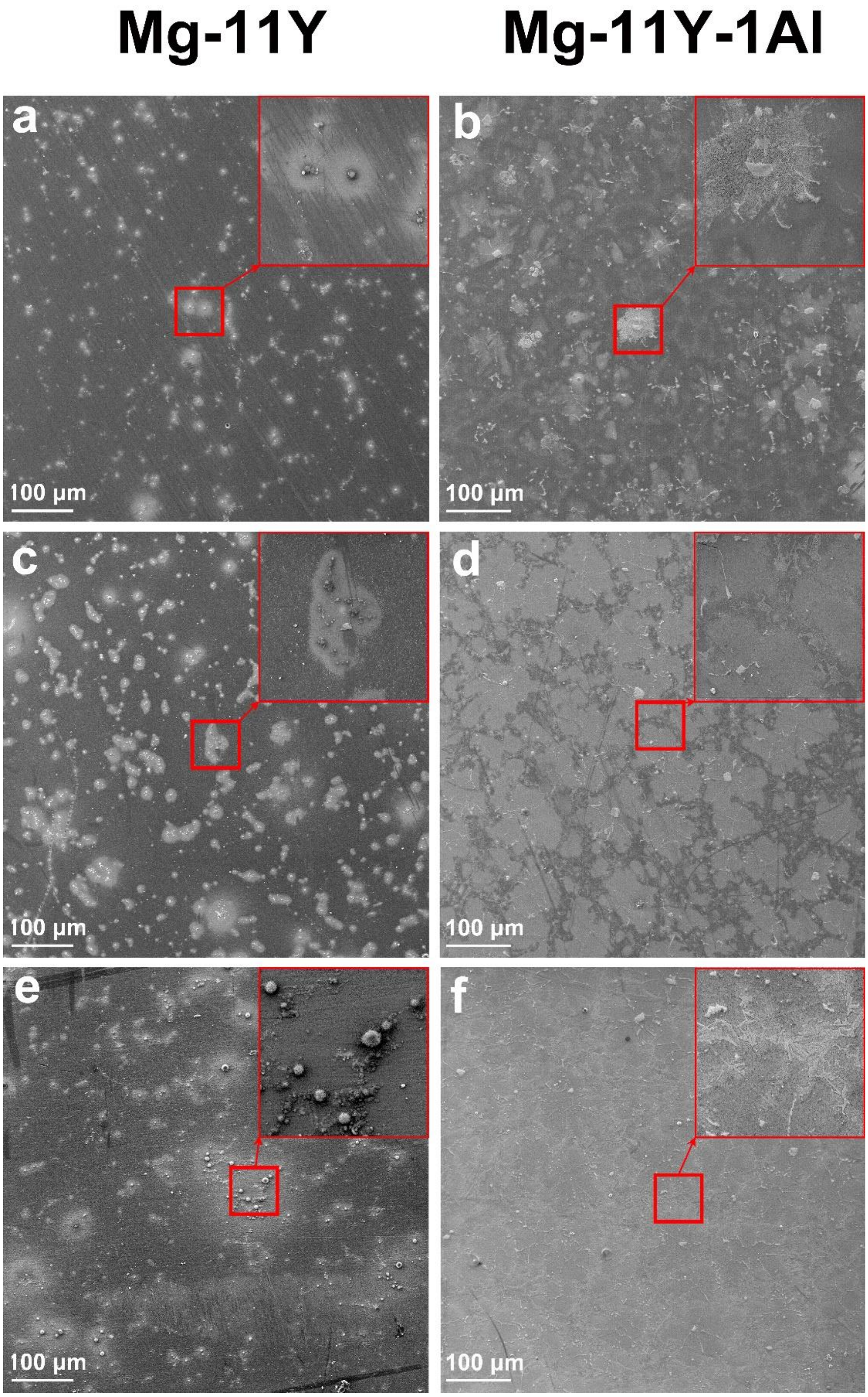

Supplementary Fig. 16 Surface morphology of W11 and WA111 alloys after soaking for a period of time. The images in the left column are as-cast W11 alloy after soaking for (a) 30 s, (c) 240 s and (e) 1200 s, respectively. The images in the right column are as-cast WA111 alloy after soaking for (b) 30 s, (d) 240 s and (f) 1200 s, respectively.

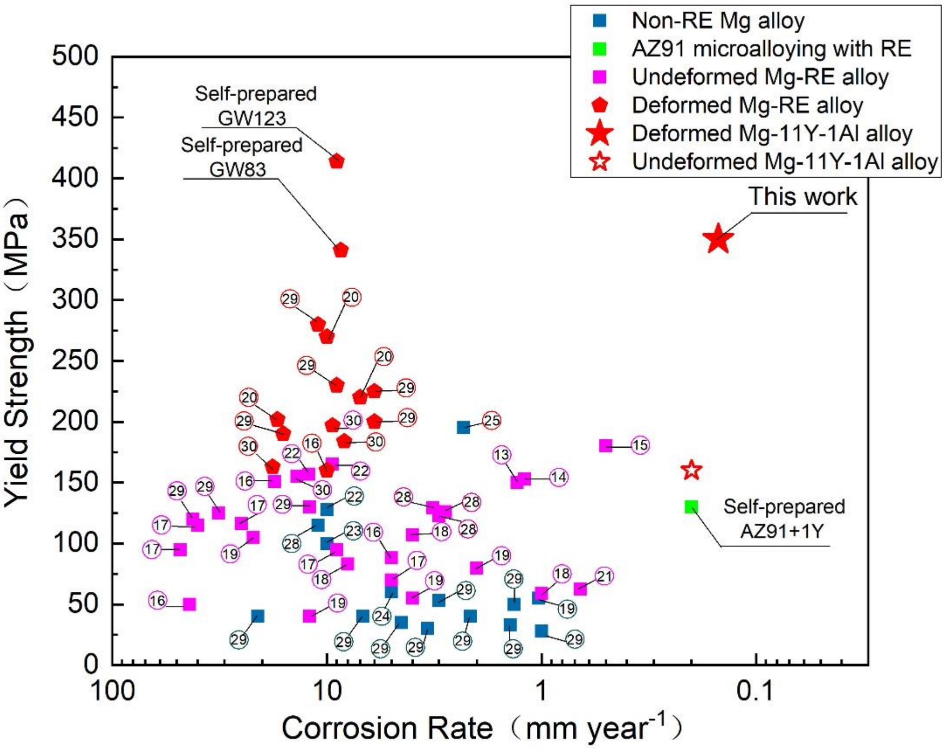

Supplementary Fig. 17 The statistical diagram of mechanical and corrosion properties of magnesium alloys prepared by conventional methods with corresponding literature sources are shown in Supplementary Fig. 17[13, 14, 15, 16, 17, 18, 19, 20, 21, 22, 23, 24, 25, 26, 27, 28, 29, 30] for Fig. 1f. The 'self-prepared' data represent some alloys mentioned in literature where either strength or corrosion rate data were not provided.

Supplementary References

1 Li YX, Qiu D, Rong YH, Zhang MX. Effect of long-period stacking ordered phase on thermal stability of refined grains in Mg-RE-based alloys. *Philosophical Magazine* **94**, 1311-1326 (2014).

2 Zhang H, Liu CQ, Zhu YM, Chen HW, Bourgeois L, Nie JF. Revisiting building block ordering of long-period stacking ordered structures in Mg–Y–Al alloys. *Acta Materialia* **152**, 96-106 (2018).

3 Qiu D, Zhang MX, Taylor JA, Kelly PM. A new approach to designing a grain refiner for Mg casting alloys and its use in Mg–Y-based alloys. *Acta Materialia* **57**, 3052-3059 (2009).

4 Qiu D, Zhang M-X. Strengthening Mechanisms and their Superposition Law in an Age-Hardenable Mg-10 wt pct Y Alloy. *Metallurgical and Materials Transactions A* **43**, 3314-3324 (2012).

5 Aagesen LK, Miao J, Allison JE, Aubry S, Arsenlis A. Prediction of Precipitation Strengthening in the Commercial Mg Alloy AZ91 Using Dislocation Dynamics. *Metallurgical and Materials Transactions A* **49**, 1908-1915 (2018).

6 Xu C, Nakata T, Qiao X, Zheng M, Wu K, Kamado S. Effect of LPSO and SFs on microstructure evolution and mechanical properties of Mg-Gd-Y-Zn-Zr alloy. *Sci Rep* **7**, 40846 (2017).

7 Somekawa H, Mukai T. Hall–Petch relation for deformation twinning in solid solution magnesium alloys. *Materials Science and Engineering: A* **561**, 378-385 (2013).

8 Yu H, Xin Y, Wang M, Liu Q. Hall-Petch relationship in Mg alloys: A review. *Journal of Materials Science & Technology* **34**, 248-256 (2018).

9 Liu D, Liu Z, Wang E. Effect of rolling reduction on microstructure, texture, mechanical properties and mechanical anisotropy of AZ31 magnesium alloys. *Materials Science and Engineering: A* **612**, 208-213 (2014).

10 Li C-j, Sun H-f, Li X-w, Zhang J-l, Fang W-b, Tan Z-y. Microstructure, texture and mechanical properties of Mg-3.0Zn-0.2Ca alloys fabricated by extrusion at various temperatures. *Journal of Alloys and Compounds* **652**, 122-131 (2015).

11 Wu Z, Ahmad R, Yin B, Sandlöbes S, Curtin WA. Mechanistic origin and prediction of enhanced ductility in magnesium alloys. *Science* **359**, 447-452 (2018).

12 Zhu Q, Shang X, Zhang H, Qi X, Li Y, Zeng X. Influence of Al2Y particles on mechanical properties of Mg-11Y-1Al alloy with different grain sizes. *Materials Science and Engineering: A* **831**, (2022).

13 Li J, Chen R, Ma Y, Ke W. Effect of Zr modification on solidification behavior and mechanical properties of Mg–Y–RE (WE54) alloy. *Journal of Magnesium and Alloys* **1**, 346-351 (2013).

14 T R, Michalska J, A K. Effect of heat treatment on corrosion resistance of WE54 alloy. *Journal of Achievements in Materials & Manufacturing Engineering* **20**, 1049-1051 (2007).

15 Chu P-W, Marquis EA. Linking the microstructure of a heat-treated WE43 Mg alloy with its corrosion behavior. *Corrosion Science* **101**, 94-104 (2015).

16 Cao F-f, Deng K-k, Nie K-b, Kang J-w, Niu H-y. Microstructure and corrosion properties of Mg-4Zn-2Gd-0.5Ca alloy influenced by multidirectional forging. *Journal of Alloys and Compounds* **770**, 1208-1220 (2019).

17 Srinivasan A, Huang Y, Mendis CL, Blawert C, Kainer KU, Hort N. Investigations on microstructures, mechanical and corrosion properties of Mg–Gd–Zn alloys. *Materials Science and Engineering: A* **595**, 224-234 (2014).

18 Li CQ*, et al.* Effect of volume fraction of LPSO phases on corrosion and mechanical properties of Mg-Zn-Y alloys. *Materials & Design* **121**, 430-441 (2017).

19 Hort N*, et al.* Magnesium alloys as implant materials--principles of property design for Mg-RE alloys. *Acta Biomater* **6**, 1714-1725 (2010).

20 Tong LB*, et al.* Microstructures, mechanical properties and corrosion resistances of extruded Mg-Zn-Ca-xCe/La alloys. *J Mech Behav Biomed Mater* **62**, 57-70 (2016).

21 Dargusch MS, Shi Z, Zhu H, Atrens A, Song G-L. Microstructure modification and corrosion resistance enhancement of die-cast Mg-Al-Re alloy by Sr alloying. *Journal of Magnesium and Alloys*, (2020).

22 Zhang C*, et al.* Effect of microalloyed Ca on the microstructure and corrosion behavior of extruded Mg alloy AZ31. *Journal of Alloys and Compounds* **823**, (2020).

23 Zhang J-l, Liu Y-l, Zhou J, Feng Z-y, Wang S-b. Kinetic study on the corrosion behavior of AM60 magnesium alloy with different Nd contents. *Journal of Alloys and Compounds* **629**, 290-296 (2015).

24 Woo SK*, et al.* Effects of combined addition of Ca and Y on the corrosion behaviours of die-cast AZ91D magnesium alloy. *Corrosion Science* **166**, (2020).

25 Wang BJ, Xu DK, Sun J, Han E-H. Effect of grain structure on the stress corrosion cracking (SCC) behavior of an as-extruded Mg-Zn-Zr alloy. *Corrosion Science* **157**, 347-356 (2019).

26 Jiang Q, Lv X, Lu D, Zhang J, Hou B. The corrosion behavior and mechanical property of the Mg–

7Y–xNd ternary alloys. *Journal of Magnesium and Alloys* **6**, 346-355 (2018).

27 Bi G, Li Y, Zang S, Zhang J, Ma Y, Hao Y. Microstructure, mechanical and corrosion properties of Mg–2Dy–xZn (x=0, 0.1, 0.5 and 1 at.%) alloys. *Journal of Magnesium and Alloys* **2**, 64-71 (2014).

28 Boby A, Srinivasan A, Pillai UTS, Pai BC. Mechanical characterization and corrosion behavior of newly designed Sn and Y added AZ91 alloy. *Materials & Design* **88**, 871-879 (2015).

29 Yin S*, et al.* Influence of specific second phases on corrosion behaviors of Mg-Zn-Gd-Zr alloys. *Corrosion Science* **166**, (2020).

30 Subasi Y, Turen Y, Zengin H, Ahlatci H, Sun Y. Effect of Zn addition on mechanical and corrosion properties of as-cast and as-extruded WE43 magnesium alloys. *Materials Research Express* **6**, (2019).

31 Liu M*, et al.* Calculated phase diagrams and the corrosion of die-cast Mg–Al alloys. *Corrosion Science* **51**, 602-619 (2009).
